# The SAGA core module is critical during *Drosophila* oogenesis and is broadly recruited to promoters

**DOI:** 10.1371/journal.pgen.1009668

**Published:** 2021-11-22

**Authors:** Jelly H. M. Soffers, Sergio G-M Alcantara, Xuanying Li, Wanqing Shao, Christopher W. Seidel, Hua Li, Julia Zeitlinger, Susan M. Abmayr, Jerry L. Workman

**Affiliations:** 1 Stowers Institute for Medical Research, Kansas City, Missouri, United States of America; 2 Department of Pathology and Laboratory Medicine, University of Kansas School of Medicine, Kansas City, Kansas, United States of America; 3 Department of Anatomy and Cell Biology, University of Kansas School of Medicine, Kansas City, Kansas, United States of America; Wayne State University, UNITED STATES

## Abstract

The Spt/Ada-Gcn5 Acetyltransferase (SAGA) coactivator complex has multiple modules with different enzymatic and non-enzymatic functions. How each module contributes to gene expression is not well understood. During *Drosophila* oogenesis, the enzymatic functions are not equally required, which may indicate that different genes require different enzymatic functions. An analogy for this phenomenon is the handyman principle: while a handyman has many tools, which tool he uses depends on what requires maintenance. Here we analyzed the role of the non-enzymatic core module during *Drosophila* oogenesis, which interacts with TBP. We show that depletion of SAGA-specific core subunits blocked egg chamber development at earlier stages than depletion of enzymatic subunits. These results, as well as additional genetic analyses, point to an interaction with TBP and suggest a differential role of SAGA modules at different promoter types. However, SAGA subunits co-occupied all promoter types of active genes in ChIP-seq and ChIP-nexus experiments, and the complex was not specifically associated with distinct promoter types in the ovary. The high-resolution genomic binding profiles were congruent with SAGA recruitment by activators upstream of the start site, and retention on chromatin by interactions with modified histones downstream of the start site. Our data illustrate that a distinct genetic requirement for specific components may conceal the fact that the entire complex is physically present and suggests that the biological context defines which module functions are critical.

## Author summary

Embryonic development critically relies on the differential expression of genes in different tissues. This involves the dynamic interplay between DNA, sequence-specific transcription factors, coactivators and chromatin remodelers, which guide the transcription machinery to the appropriate promoters for productive transcription. To understand how this happens at the molecular level, we need to understand when and how coactivator complexes such as SAGA function. SAGA consists of multiple modules with well characterized enzymatic functions. This study shows that the non-enzymatic core module of SAGA is required for *Drosophila* oogenesis, while the enzymatic functions are largely dispensable. Despite this differential requirement, SAGA subunits appear to be broadly recruited to all promoter types, consistent with the biochemical integrity of the complex. These results suggest that genetic requirements for different modules depend on the developmental demands.

## Introduction

The Spt/Ada-Gcn5 Acetyltransferase (SAGA) complex is required for the transcription of most RNA polymerase II (Pol II) genes [1]. It contains several multi-protein modules that have specialized functions in transcription. The histone acetyltransferase (HAT) module binds histone (H) 3 lysine (K) di- and trimethylated histones and preferentially acetylates H3K9 and H3K14, which leads to gene activation [2–5]. The deubiquitinase (DUB) module removes ubiquitin from H2B. H2Bub is involved in regulating transcription elongation and regulates further histone modifications [6,7]. The other two modules have no described enzymatic function. The module formed by the large Nipped-A subunit accommodates interactions with transcription activator proteins, which are important for promoter recruitment [8]. The core module is composed of Suppressor of Ty subunits Spt3 and Spt20, Ada1 and several TBP-associated factor (TAF) subunits. Some of the TAFs are shared with the promoter recognition complex TFIID, and both complexes bind TATA Binding Protein (TBP) [9]. The structural basis for TBP binding is highly conserved between the SAGA complexes across species and depends on interactions with Spt3 [10–13]. TBP binds DNA in a sequence-specific manner and helps to position the preinitiation complex.

The modules form a stable biochemically entity, and it has been reported that module functions are linked [14]. Thus, one would expect that modules function synergistically to activate transcription, and that disruption of any part disrupts the functionality of the entire complex. Paradoxically, work in *Drosophila*, yeast and mammals indicates that the disruption of different modules has different effects on gene regulation. Our previous work demonstrated that oogenesis requires the SAGA HAT module function, but not the DUB module function, indicating that not all genes require all enzymatic functions simultaneously [15,16]. An analogy to this is the handyman principle: while a handyman has many tools, he will hardly use them all at the same time, and which tool he uses will depend on what requires maintenance. This raises the possibility that distinct gene sets require different functions of the complex in defined biological contexts. The critical question therefore becomes what the exact functions are of each module during gene regulation, and what gene features define the dependence on one or more functions of the SAGA complex.

We took advantage of the genetic model system of *Drosophila* oogenesis to study the precise functions of the SAGA core module in promoter regulation [16–18]. The SAGA core module interacts with TBP. TBP and the structurally related protein TBP-related factor 2 (TRF2) play critical, yet different roles during spermatogenesis and oogenesis [19–23]. Thus, we hypothesized that the core module is important for oogenesis via the activation of a distinct gene set, in a way that depends on TBP. We specifically targeted subunits of the *Drosophila* SAGA core complex that are not shared with TFIID: SAGA factor like TAF6 (SAF6) [24], TAF10b [25] and Will Decrease Acetylation (WDA) [14]. We found that depletion of these SAGA-specific TAFs arrested egg chamber development during mid-oogenesis, demonstrating a critical role for the SAGA core module. Depletion of the TBP-binding core subunit Spt3 phenocopied this oogenesis defect, suggesting that this function is dependent on TBP. The direct depletion of TBP or TFIID subunits resulted in a more severe phenotype, indicating that the latter play a broader role. The genome-wide occupancy of SAGA subunits, as observed in ChIP-seq experiments or in the higher resolution ChIP-nexus experiments, showed that SAGA subunits co-occupied all promoter types and correlated with transcriptional activity, but did not indicate that the SAGA complex specifically associates with TBP or TRF2 at distinct promoter types. However, at high resolution, differences in binding patterns between promoter types became apparent. The complex preferentially binds either upstream of the start site, where activators are found, or downstream of the start site, where SAGA may be retained at the +1 nucleosome.

In conclusion, we report that during oogenesis, the non-enzymatic core module functions are most critical. Its critical role cannot be explained by an exclusive recruitment of this module as an isolated complex or even the canonical SAGA complex to distinct promoter types. Together, these findings suggests that the SAGA complex binds globally to active genes, but that its module-specific activities are differentially required, such that it regulates distinct genes in a module-specific fashion. Which module becomes critical depends on what genes need to be expressed, and this is defined by the biological context.

## Results

### SAGA modules have distinct roles during Drosophila oogenesis

Oogenesis in *Drosophila* occurs in an assembly line fashion in units called ovarioles, which are strings of six or seven sequentially developing egg chambers. The development of the egg chambers is divided into early stages (1–6), mid (stage 7-10a) and late stages (10b-14) [26]. During mid-oogenesis, the egg chambers take on an elongated shape and the oocyte increases in size.

**Fig 1:**
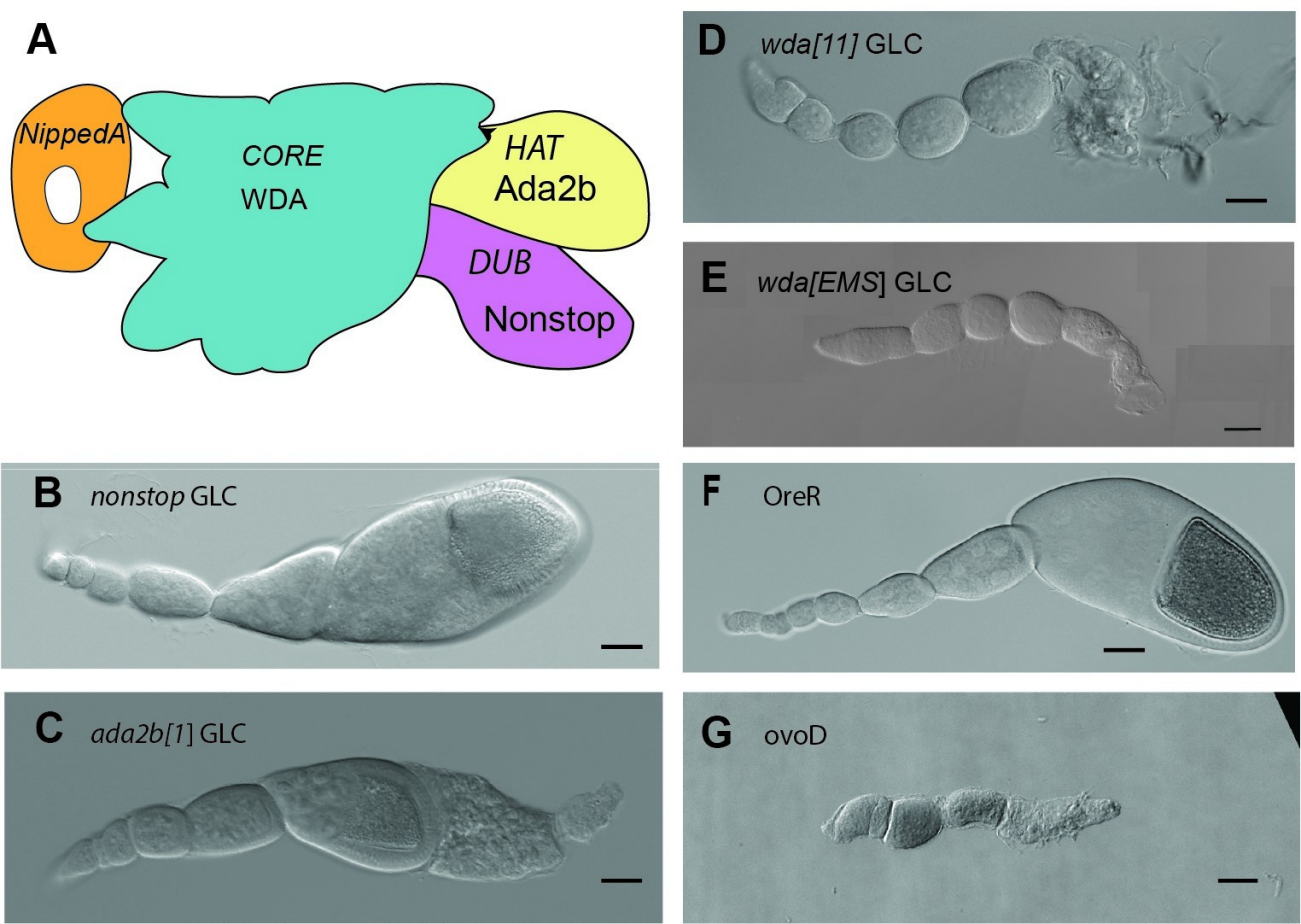
Different requirements of the SAGA modules during oogenesis. (A) Cartoon of the SAGA complex. (B-G) Differential contrast images of GLC ovarioles and controls. Loss of the enzymatic functions leads to no or a late defect in oogenesis, because DUB subunit Nonstop is dispensable, whereas the *ada2b[1]* GLC ovaries display a stage 12 defect. (B) *Nonstop* GLC ovariole. (C) *Ada2b[1]* GLC ovariole. (D) *wda[11]* ovariole. (E) *wda[EMS]* ovariole. (F) OreR wild type control ovariole. (G) ovoD control ovariole. Scale bar: 50 μM.

The SAGA enzymatic and core subunits are found in different modules of the complex (Fig 1A). We reported previously that the enzymatic components of SAGA are differentially required for oogenesis [15]. The DUB module is dispensable for oogenesis since mature eggs form after the depletion of the enzymatic deubiquitinase subunit Nonstop in the germ line (Fig 1B). Ovaries lacking Ada2b (Fig 1C), the subunit that modulates Gcn5 acetyltransferase function, display defects in late oogenesis stages. These *ada2b[1]* germ line clone (GLC) ovaries still have enlarged oocytes typical of late stages, but the dorsal filaments fail to form and the oocytes degenerate before they develop into mature eggs (Fig 1C).

To test the genetic requirement of the core module, we depleted the SAGA-specific core subunit WDA in the germ line cells by generating GLC of *wda[11]* (Fig 1D) [27]. Since the *wda[11]* allele also disrupts a neighboring gene [14], we performed an ethyl methanesulfonate (EMS) mutagenesis screen to isolate a new mutant allele of *wda* (*wda[EMS])* (Fig 1E). In *wda[11]* or *wda[EMS]* GLC ovaries, the number of round early-stage egg chambers increased and late-stage egg chambers did not form, indicating a failure to progress through stage 7 of mid-oogenesis. WDA is an integral part of the core module, and one could argue that this defect is caused by the loss of complex integrity, which would result in a failure to link the enzymatic modules. However, further deletion of two key structural core subunits Spt20, Spt7 [28–30] by germ line-specific RNA interference (RNAi) did not phenocopy this defect (S1 Fig), suggesting that the defect is not caused by the loss of a scaffold function for the enzymatic modules. Moreover, since the DUB module is dispensable for oogenesis and the loss of Ada2b only becomes problematic at late stages, these data suggest that this phenotype is not mediated by a loss of catalytic functions. Together, these experiments uncovered an important and early function for the SAGA core module during oogenesis.

### SAGA core subunits are critical for mid-oogenesis

**Fig 2:**
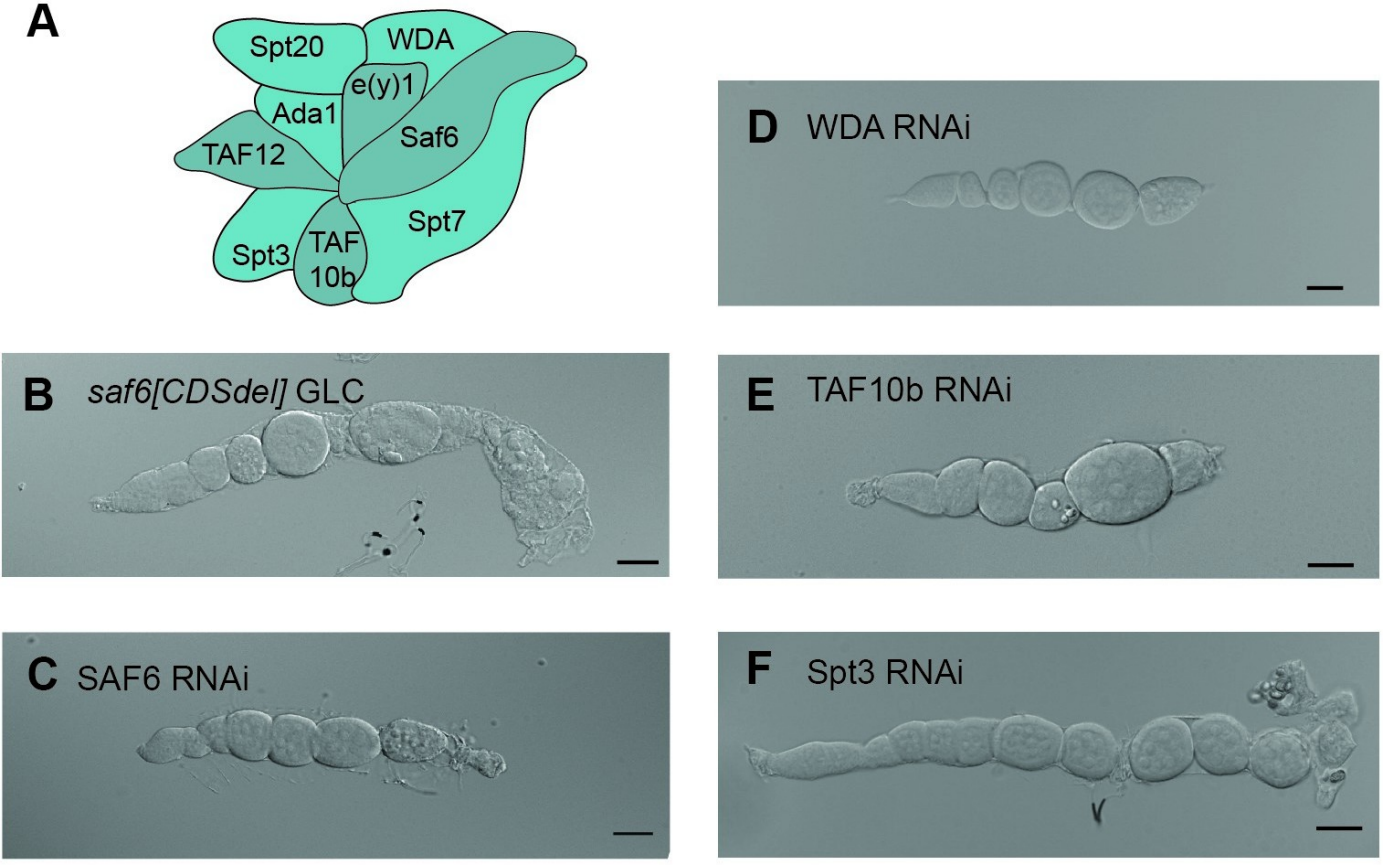
The core module of the SAGA complex is required for mid-oogenesis. (A) Overview of SAGA core subunits. (B) *saf6[CDSdel]* ovariole. (C) SAF6 RNAi ovariole. (D) WDA RNAi ovariole. (E) TAF10b RNAi ovariole. (F) Spt3 RNAi ovariole. Scale bar: 50 μM.

To corroborate the role of the core module during oogenesis, we depleted the other SAGA-specific core subunits in the germ line and asked whether this phenocopies the WDA oogenesis defect (Fig 2A). We created a *saf6* deletion allele by replacing the coding region with a dsRED cassette using CRISPR Cas9 (see Materials and Methods) and generated GLC ovaries with this allele (Fig 2A). Loss of SAF6 (Fig 2B) led to a phenotype like the loss of WDA (Fig 1D and 1E). The egg chambers failed to elongate but remained round, and the oocyte did not increase in size, suggesting that oogenesis arrested at stage 6. A similar phenotype was observed when SAGA-specific TAF subunits were targeted in the germ line by RNAi. The depletion of SAF6, WDA or TAF10b all arrested oogenesis at stage 6–7 with similar phenotypes (Fig 2C–2E), demonstrating that SAGA-specific TAF subunits are critical for the progression through mid-oogenesis.

### SAGA core subunits may function through TBP

Based on the homology with yeast SAGA complexes, we infer that WDA interacts with SAF6 and TAF10b, which are important for the structural configuration of Spt3 that allows the binding of TBP. Therefore, we hypothesized that the SAGA-specific TAF subunits are critical for oogenesis because they mediate the ability to bind TBP. To test this, we targeted Spt3 by RNAi in the germline. This also arrested oogenesis at stage 6–7 and produced a phenotype highly reminiscent of those observed for the TAFs (Fig 2F). The number of immature egg chambers increased, but they remained round, and the oocyte did not enlarge at the distal end. This result suggests that the SAGA-specific TAF subunits are required for the function of Spt3 and thus likely influence the deposition of TBP onto promoters during oogenesis.

**Fig 3:**
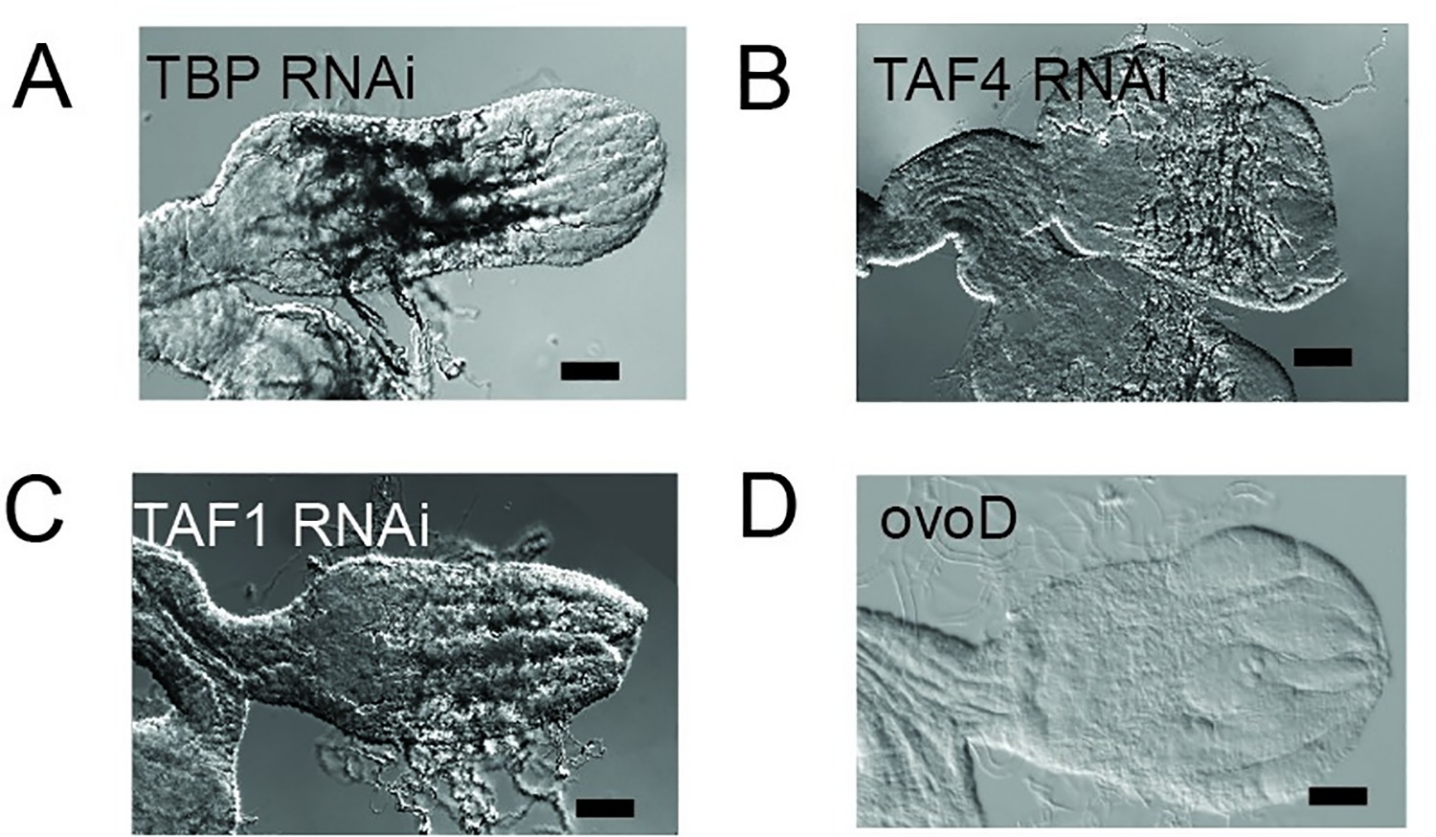
TFIID subunit depletion blocks oogenesis earlier than SAGA subunit depletion. (A) TBP RNAi ovary pair (B) TAF4 RNAi ovary pair. (C) TAF1 RNAi ovary pair. (D) OvoD control ovary pair. Scale bar: 50 uM.

We next tested whether the direct depletion of TBP in the germ line caused a similar phenotype. However, germ line depletion of TBP led to a more severe phenotype (Fig 3A), indicating that other TBP-containing complexes function in the germ line. To test this, we depleted TAF4 (Fig 3B) and TAF1 (Fig 3C), which are TFIID-specific TAF subunits. Depletion of TAF4 and TAF1 with RNAi all caused severe agametic phenotypes, confirming that TBP and TFIID play a broader role during oogenesis than the SAGA complex. This result agrees with previous work that demonstrated that a smaller gene set is affected by loss of SAGA versus TFIID [31].

### The SAGA complex broadly binds to various promoter types

The stronger phenotype associated with depletion of TBP compared to SAGA-specific core subunits prompted us to test whether the role of SAGA is specific for certain promoter types. Such distinction is of particular interest in a developmental context, where promoter types may be preferentially used [32,33]. For example, TATA promoters in *Drosophila* are enriched amongst the earliest expressed developmental genes in the embryo [34]. The phenotype of Spt3 depletion pointed to a connection between SAGA and TBP in the ovary. We hypothesized that SAGA regulates a subset of TBP-bound TATA genes, and expected that a large proportion of SAGA- regulated genes would contain TATA promoters.

We first determined how SAGA occupies promoters in ovary tissue genome-wide. We performed chromatin immunoprecipitation followed by sequencing (ChIP-seq) using antibodies against the SAGA core module subunits WDA, SAF6 and Spt3, and against Ada2b, a SAGA HAT module subunit. We then compared their occupancy patterns and analyzed how they relate to the promoter activities. As measurement for each promoter’s activity, we used Cap Analysis Gene Expression (CAGE) data from ovaries of virgin females (modENCODE_5368 [35]), which yielded capped transcripts for approximately 6,000 promoters (> 2 TPM), and Pol II occupancy data [36].

**Fig 4:**
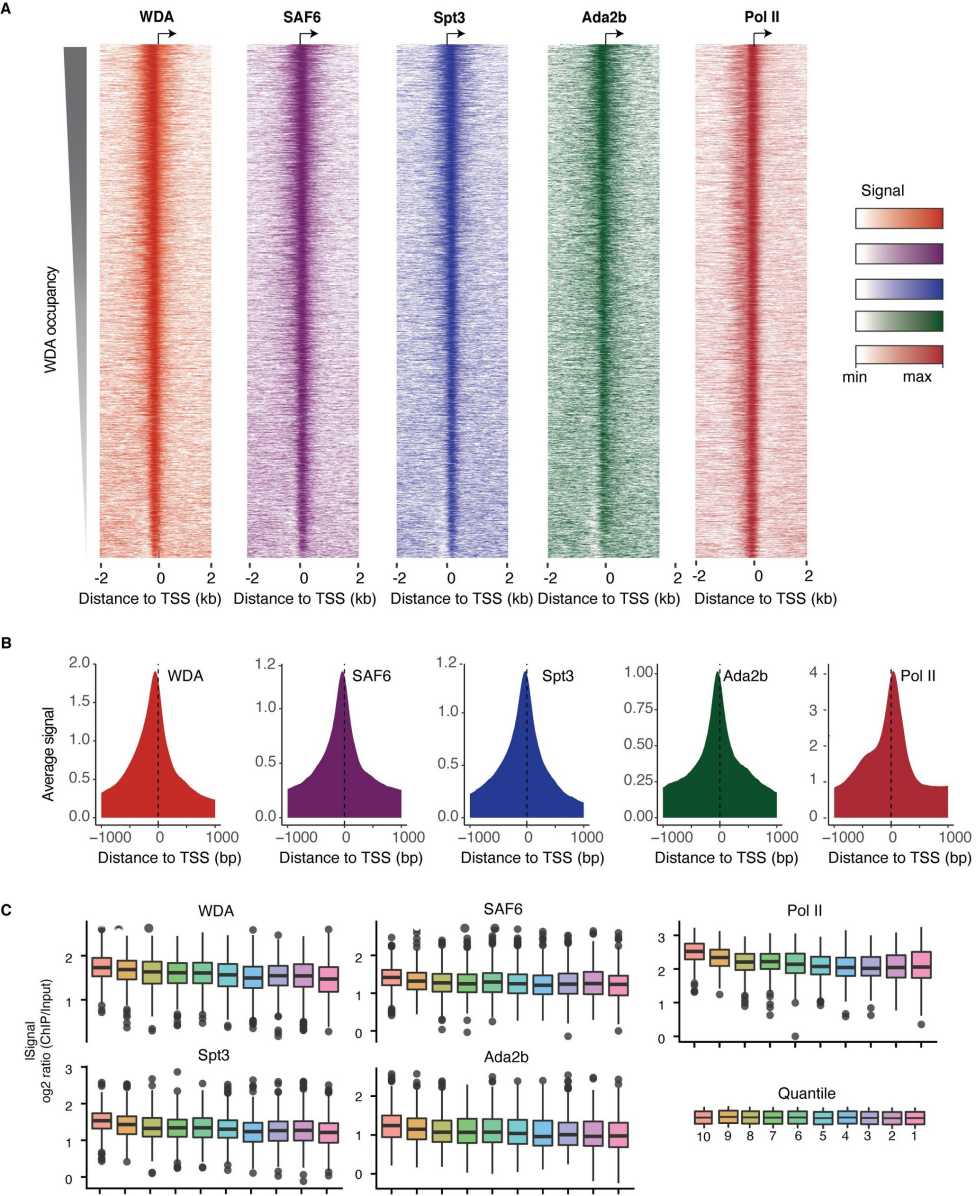
Genome-wide occupancy of SAGA subunits in ovary tissue shows that SAGA broadly binds to active promoters. (A) Normalized ChIP signal in a 1001 bp window centered at the TSS of active genes sorted by WDA levels. (B) Average binding of SAGA subunits and Pol II at all active genes. (C) Active genes were divided into ten quantiles based on CAGE expression levels (decreasing from left to right) and each box plot panel displays the SAGA subunit occupancy per quantile (log2 ratio over input). This shows that SAGA occupancy correlates moderately with transcriptional activity.

Our data revealed widespread occupancy of all SAGA subunits at active promoters, as well as a strong correlation between the different subunits. When we sorted the top 4,000 active promoters by WDA occupancy, the occupancy pattern was visually very similar for the other subunits (Fig 4A). For all subunits, the average binding pattern was strongest near the transcription start site (TSS), and followed the distribution pattern of Pol II, supporting its function at the core promoter (Fig 4B). To investigate the relationship between occupancy and transcription, we grouped all active genes into ten quantiles of CAGE expression levels and calculated within each quantile the binding levels of SAGA at each promoter. With decreasing expression levels, we observed a gradual decrease in occupancy for all examined SAGA subunits (Fig 4C). A similar correlation was observed using RNA-seq (S2 Fig). Together, these results show that SAGA binding in the ovaries is widespread at active promoters and suggest that the degree of occupancy correlates with transcriptional activity.

We next analyzed whether SAGA regulated a distinct promoter type by analyzing the ChIP-seq occupancy data in the ovary and intersecting this with different categories of active genes. We analyzed the following *Drosophila* promoter types: 1) TATA promoters (TATA), 2) promoters with Downstream Promoter Region elements (DPE), 3) promoters with the polypyrimidine initiator element TCT (TCT), and 4) housekeeping (HK) promoters that may contain a DNA replication-related (DRE) element or Ohler motifs 1, 6 and 7 (but lack TATA, TCT or DPR elements) [37–39]. TCT promoters require TBP Related Factor 2 (TRF2) and not TBP for their activation [40]. TRF2 is structurally similar to TBP but lacks high affinity binding for TATA boxes [19] and binds to different sites in the *Drosophila* germ line [22]. In addition, TRF2 interacts with DNA replication-related factor (DREF), the factor that binds to the DRE motif [22,41]. We identified 156 TATA genes, 341 DPE genes, 102 TCT genes and 1285 HK genes among the active promoters with CAGE-seq (S3 Fig). We further found that SAGA occupancy was not higher at TATA promoters or lower at promoters that preferentially use TRF2, further arguing against promoter-specific recruitment of SAGA in the ovary (S4 Fig).

**Fig 5:**
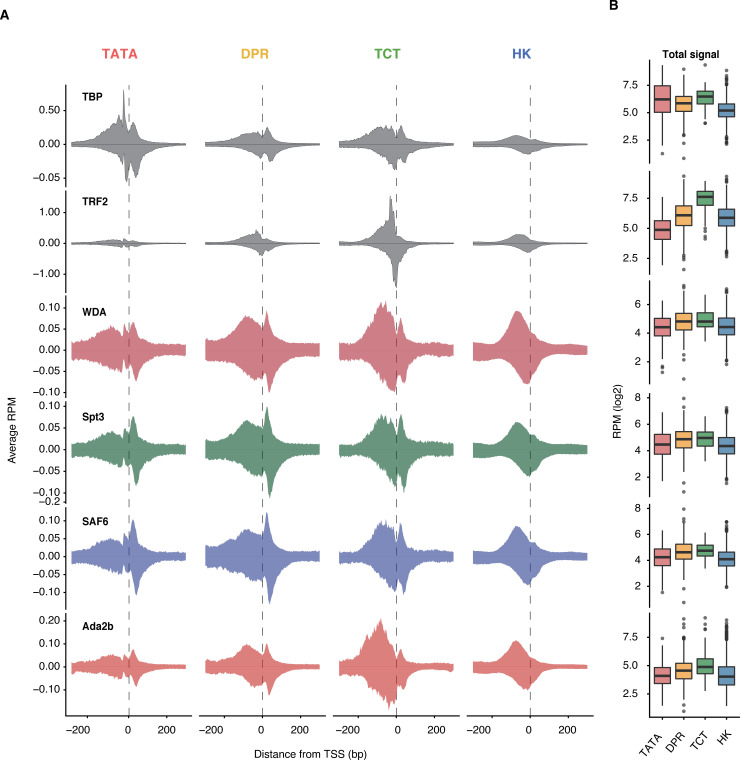
SAGA footprint at different promoter types in cells. **(**A) Average footprints of SAGA subunits in reads per million (RPM) (left to right TATA, DPE TCT, HK). From top to bottom TBP and TRF2, SAGA subunits WDA, Spt3, SAF6 and Ada2b. (B) SAGA levels are overall similar across promoter types.

ChIP-seq data are however of low resolution and may capture unspecific binding. In contrast, our established ChIP-exo protocol called ChIP-nexus has higher specificity and near base-resolution [42]. It has previously revealed the exact locations of pre-initiation complex components along the promoter, including TBP [43]. Since we could not perform ChIP-nexus experiments in ovaries, we used *Drosophila* Kc167 cells and performed ChIP-nexus experiments against the four SAGA subunits, as well as against TBP and TRF2 (Figs 5 and S5). To analyze their binding profile for each promoter type, we identified 164 TATA genes, 320 DPE, 103 TCT genes and 1385 HK genes among the active promoters that were identified using Kc167 CAGE-seq data (S5 Fig). As expected, the highest levels of TBP were observed at TATA promoters (Fig 5). TRF2 had not previously been analyzed with ChIP-nexus, but the high levels of TRF2 at the TCT and HK promoters were consistent with previous ChIP experiments and functional studies [40,41]. In contrast to TBP and TRF2, the four SAGA subunits appeared to be enriched at comparable levels across all four promoter types like in the ovary tissue (S4 Fig). Thus, the SAGA complex does not to appear to bind a specific promotor type.

However, at this level of resolution, it became apparent that there are slight differences in the SAGA binding patterns between promoter types. Each SAGA subunit binds certain locations upstream and downstream of the TSS with variable amounts. For example, SAGA subunits bound mainly upstream of the TSS at TCT and housekeeping genes, but appeared more prominent downstream of the TSS at TATA and DPR genes (Fig 5). Since SAGA is recruited by activators, this difference could be due to the different location of the enhancer regions. These are located directly upstream of the TCT and housekeeping promoters, but are found more distally at TATA and DPE genes and thus would not contribute to the signal in the average profile around the promoter. While these profiles with upstream and downstream binding were interesting, they did not support our hypothesis that SAGA is specifically associated with TBP at TATA promoters.

### SAGA binds TBP robustly but was not found to bind TRF2

**Fig 6:**
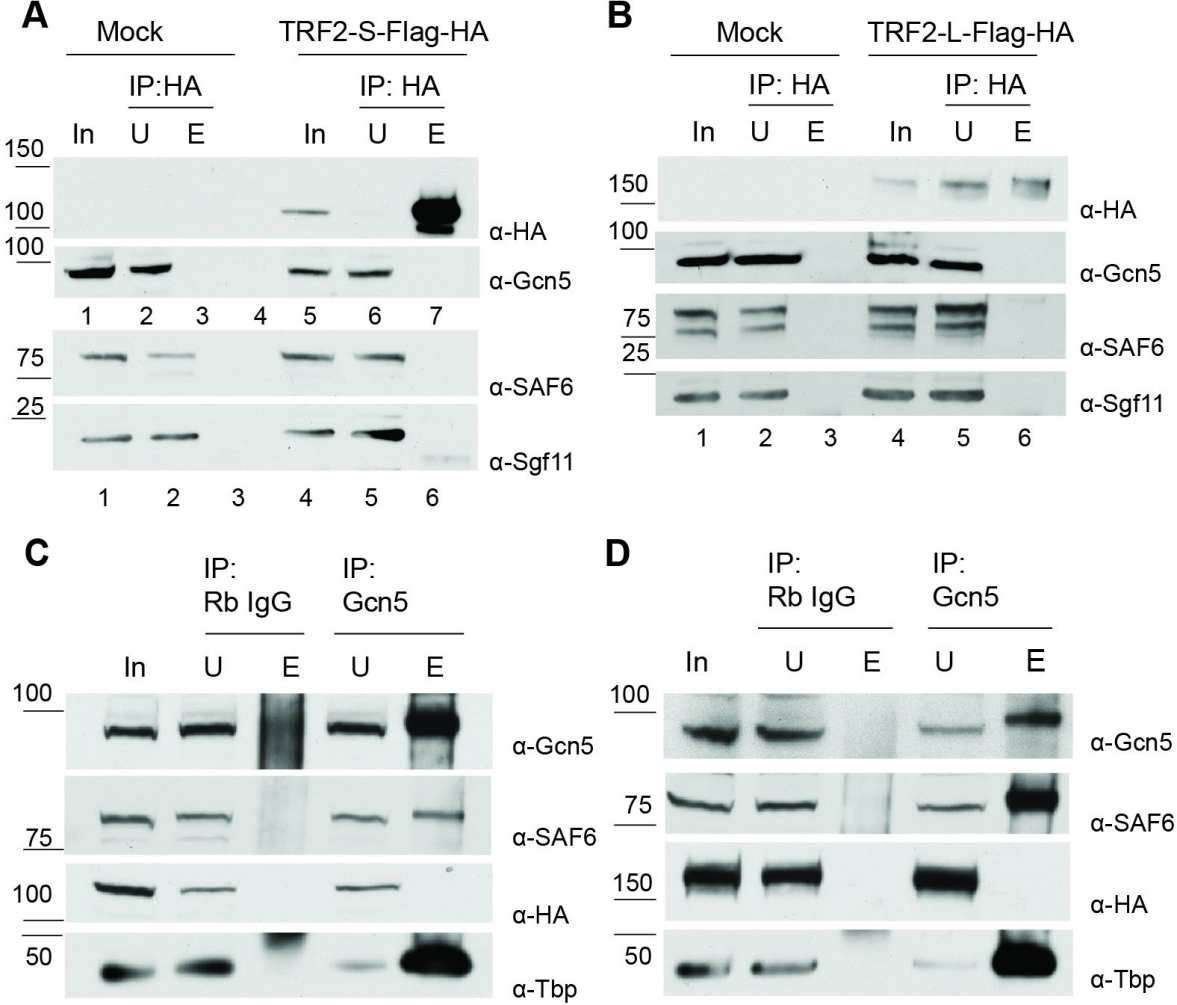
SAGA and affinity-tagged TRF2 isoforms do not stably interact in S2 cells. (A) Reciprocal coimmunoprecipitation after transient transfection with pAcTRF2-S-Flag-HA. Using rat anti-HA to immunoprecipitated TRF2-S-Flag-HA, SAGA did not coimmunoprecipitate. (B) Reciprocal coimmunoprecipitation after transient transfection with pAcTRF2-L-Flag-HA. Using rat anti-HA to immunoprecipitated TRF2-L-Flag-HA, SAGA did not coimmunoprecipitate. (C) Coimmunoprecipitation after transient transfection with pAcTRF2-S-Flag-HA. Using Gcn5 as a bait, affinity-tagged TRF2-S does not coimmunoprecipitate. (D) Coimmunoprecipitation after transient transfection with pAcTRF2-L-Flag-HA. Using Gcn5 as a bait, affinity-tagged TRF2-L does not coimmunoprecipitate. In: unbut U: unbout E: eluate.

Since SAGA was strongly bound at TCT genes, where TRF2 functions, we considered that SAGA may also be associated with TRF2. This was of particular interest in relation to our phenotype, which suggested a problem with the endocycle in the germ line. The switch in the endocycle forms a checkpoint at the transition from early to mid-oogenesis, such that its timing coincides with the core module phenotype. Proper regulation of the endocycle in somatic ovary cells requires TAF9, which is a core module subunit that SAGA shares with TFIID [44–47]. TRF2 is reported to bind critical cell cycle genes [48] and cell cycle speed has been implicated with distinct promoter regulation during development [32]. Because the structure of TRF2 is similar to that of TBP and could theoretically bind SAGA, we tested if the SAGA complex stably interacts with TRF2 by co-immunoprecipitation. We transiently transfected epitope-tagged constructs in S2 cells. We tested both TRF2 isoforms since both form higher molecular weight complexes [22,41]. However, we did not detect SAGA subunits after immunoprecipitation of TRF2 (Fig 6A and 6B). Likewise, we failed to detect TRF2 isoforms after immunoprecipitation of the SAGA complex (Fig 6C and 6D). As control, endogenous TBP interacted robustly with SAGA subunits (Fig 6C and 6D). These results indicate that epitope-tagged TRF2 and SAGA do not stably interact, consistent with similar observations in murine testes, where TRF2 co-immunoprecipitated with TFIIA but not TAF7L, although it co-localized in ChIP experiments with both [49]. It is therefore possible that the colocalization of SAGA and TRF2 *in vivo* requires a chromatin template.

## Discussion

SAGA is a large, versatile coactivator complex in which all subunits and modules typically tightly interact with each other biochemically. However, although the entire complex is known to bind to a large proportion of the *Drosophila* genome, only certain genes depend on its activity for their gene activation, and moreover, different genes depend on the functions of different enzymatic modules in a given biological context (reviewed in [8]).

Until now, it was not clear if the SAGA complex is required for oogenesis. Previous studies demonstrated that the SAGA DUB module is dispensable for oogenesis, but that a histone acetylation activity mediated by Gcn5 and Ada2b is critical for the progression of oogenesis [15,16]. Because this HAT activity could be accounted for by the ADA or Chiffon complexes [17,18], these results did not establish an unequivocal role for the SAGA complex during oogenesis. Our present genetic analysis demonstrated that the depletion of the subunits that are specific to the SAGA core module arrested egg chamber development during mid-oogenesis. Therefore, the SAGA complex is critical for gene regulation during oogenesis.

Moreover, it showed that not all modules are equally important for oogenesis. In the remainder of this study, we evaluated if promoter structure could account for such differential requirement. The differential genetic requirements of subunits or modules have sometimes been interpreted as the result of their differential recruitment to specific genes. Especially during development, differential promoter usage appears to be a key regulatory mechanism [15,23,32, 33,50,51]. We found that SAGA bound to the majority of the transcribed genes as canonical complex. Moreover, we observed that the active SAGA-bound genes were not restricted to distinct promoter types. These results were somewhat surprising, since our genetic and biochemical data suggest a specific interaction of SAGA with TBP, but not TRF2. However, these results strengthen the more recently established role of SAGA as general transcription coactivator [52,53] and support the idea that the different functions of SAGA are not explained by the complex being recruited to specific promotor types. However, we cannot exclude that under depletion conditions, SAGA recruitment patterns differ and cause these distinct oogenesis defects. Moreover, we evaluated the SAGA binding pattern in an ovary homogenate, and this might obscure possible differences in binding patterns between somatic and germ cells. We therefore cannot rule out that SAGA coactivation activities differ between promoter types in the germ line. We attempted to analyze this by comparing transcript levels between mutant and wild type ovarioles, but the high transcript levels generated by the highly abundant wild-type somatic cells and the presence of more mature egg chambers in the wild-type ovaries made this analysis inconclusive. Therefore, we were not able to determine which genes change expression in the germ line cells within stage 1–6 egg chambers upon the loss of SAGA core module function.

**Fig 7:**
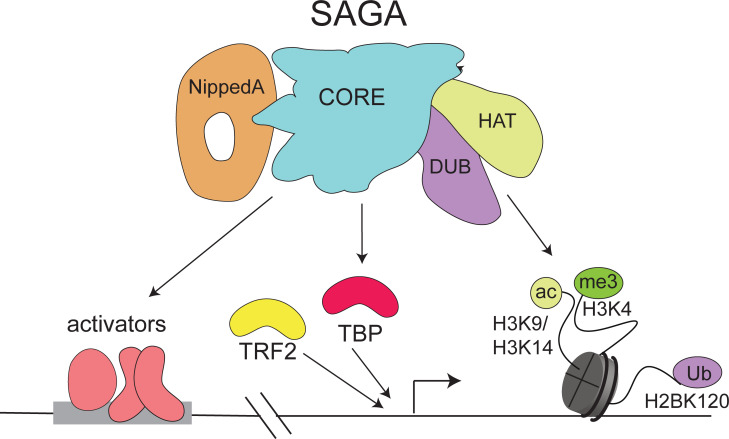
Cartoon of SAGA core module functions during oogenesis. The SAGA complex binds upstream and downstream of the TSS at active genes, without discriminating by promoter type. The upstream peak is likely due to interactions of the core module and Nipped A, which interact with activators. The complex is also present at the +1 nucleosome, where it can read H2bUb, H3K4me3 and H3K9/4ac and is retained. After its recruitment to the +1 nucleosome, the enzymatic DUB and HAT activities of SAGA retain the complex, carryout histone modifications and may help maintain the core promoter region in an open state, while the core module may contribute to deposition of TBP onto the core promoter. We speculate that through its capacity to interact with TBP, the SAGA core module may help to increase the concentration of TBP near promoter chromatin.

Nonetheless, the genetic analysis pointed to functional differences among the modules, which suggests that the SAGA complex functions as handyman. During oogenesis the core module’s promoter function is most critical, although all modules are recruited to all active promoters. This could be because of its interaction with TBP, which may help position TBP upstream of the transcription initiation site, although SAGA may also function at promoters that primarily use TRF2 for activation (Fig 7). Another explanation for the critical requirement of the core module is that it mediates the recruitment of other SAGA modules to chromatin, and that their joint connection and putative crosstalk is important for proper gene regulation. The recruitment and activity of SAGA depends on activator proteins [54]. The core module links Nipped-A (yeast Tra1), the major hub for activator interactions, to the enzymatic modules. Nipped-A is critical for gene regulation in early states of *Drosophila* oogenesis [55]. The core module itself also contains subunits directly involved in promoter binding [56]. Consistent with this idea, histone acetylation by the SAGA HAT module depends on promoter recruitment [2,57] and this requires certain core module subunits in *Drosophila* [14].

Our high-resolution ChIP-nexus data provided additional insights into the interaction of SAGA subunits with chromatin. While previous Drosophila SAGA complex ChIP data only showed broad associations with promoters and RNA polymerase II [15,58], ChIP-nexus detected preferential binding in regions upstream of the start site, where activators are found [38], consistent with the role of activators in SAGA recruitment. In addition, the ChIP-nexus data revealed binding of SAGA downstream of the start site, near the +1 nucleosome. This raises the interesting possibility that SAGA binding may be stabilized through its interaction with nucleosomes. Several biochemical studies support this possibility. The bromodomains within the catalytic subunits of SAGA interact with acetylated histones and the tandem tudor-domains within the HAT module serve as H3K4me3 readers [59], whose levels depend on H2Bub, to further stimulate the processive acetylation by SAGA [60,61]. Thus, there appears to be extensive crosstalk, in which SAGA’s ability to recognize its own histone modifications may promote further interactions with nucleosomes. Given this crosstalk, we propose a model in which SAGA’s occupancy at promoters is in part due to the recruitment via activators, and in part due to its retention on chromatin via modified nucleosomes.

## Materials and methods

### Antibody production

Affinity purified antibodies for ChIP analysis were generated by the GenScript Company following the PolyExpress Premium Antigen-Specific Affinity Purification Production protocol. Antigens included full length Gcn5, WDA and SAF6.

### Fly housing and ovary dissection

*Drosophila* melanogaster were raised and maintained at 25°C with a 12 hour/12 hour, light/dark cycle in non-crowding conditions. The animals were reared at 25°C on standard fly food containing 14 g inactive dry yeast (Genesee Scientific, Cat. Num. 62–108), 46 g yellow corn meal (VWR, Cat. Num. 75860–346), 6.9 g agar (Genessee Scientific, Cat. Num. 6–103), 11.5 mL corn syrup (Reinhart Food Service, Cat. Num. 31494), 53.5 g dried malt (Briess Industries Inc, Cat. Num. 5728), 8mL 30% Tegosept (Genesee Scientific, Cat. Num. 20–258) in ethyl alcohol (Sigma-Aldrich, Cat. Num. 459836), 6 mL propionic acid (Sigma-Aldrich, Cat. Num. 402907), and 900 mL of water. Ovaries were dissected in M3 Shields and Sang media from females conditioned three days on apple juice plates supplemented with yeast paste. A list of genotypes is included in S1 Table.

### Cell culture and transient transfections

Wild type S2 or Kc167 S2 cell lines were maintained at 1–2 e^6^ cells/mL in SFX medium (HyQ SFX-Insect;HyClone Laboratories, Inc) at 27°C. Transient Transfections of S2 cells were performed using Qiagen Effectene Transfection reagent (Qiagen 30425) at a 1:25 DNA-Enhancer ratio. With respect to cell density and culture volume, 3e^6^ S2 cells resuspended in 2.5 mL SFX medium were transfected with 0.4 μg plasmid DNA and this was scaled up when required. pAcTRF2-S-Flag-Ha and pAcTRF2-L-Flag-Ha were kindly gifted by the Gershon laboratory [37].

### Coimmunoprecipitation

Nuclear extracts of 15 e^7^ S2 cells were prepared five days post-transfection. Cells were collected by 1 800 x g centrifugation for five minutes at 4°C. Cells were next resuspended in 1mL lysis buffer (10 mM HEPES [pH 7.9], 1.5 mM MgCl_2_, 10 mM KCl and 1% NP-40) supplemented with 1mM PMSF (Millipore Sigma) and 1x Roche cOmplete Protease Inhibitor Cocktail Tablets (Millipore Sigma). After five minutes lysis on ice the nuclei were collected by centrifugation at 10 400 x g for five minutes at 4°C. The nuclei pellet was incubated with 0.5 mL high-salt Nuclear Extraction buffer (20 mM HEPES [pH 7.9], 25% glycerol, 1.5 mM MgCl_2_, 0.2 mM EDTA, 0.1% Triton- X100 and 420 mM NaCl) supplemented with 5 μl benzonase (Millipore Sigma), 1mM PMSF and 1X protease inhibitor for one hour at 4°C while rotating. The soluble nuclear proteins were collected as supernatant after centrifugation at 15 000 RPM for 20 minutes at 4°C. Protein concentrations were measured by Bradford assay.

Immunoprecipitations were performed with 1.5 mg nuclear extract diluted to 0.15 M NaCl with Dignam buffer. Each IP reaction was performed with 50 μl Dynabeads precoated with 1 μg of protein. The capturing antibody (rabbit FL Gcn5 (Genscript), rat high affinity anti HA (MilliporeSigma 11867423001)) or IgG (Santa Cruz) were incubated with 50 μl washed Dynabeads resuspended in 1 mL PBS (A for rabbit Invitrogen 10001D/, G for rat antibodies Invitrogen10003D). After binding for two hours at 4°C while rotating, unbound bait protein was removed, and the beads were washed one time in 1mL cold PBS prior to incubation with the nuclear lysate for immunoprecipitation. The next morning, the unbound extract was collected, and the beads were washed three times in 1 mL wash buffer (10 mM HEPES [pH 7.9], 1.5 mM MgCl_2_, 150mM NaCl, 10 mM KCl, 0.2% Triton X-100) prior to elution in 100μl 2x Laemmli Sample buffer (Bio-Rad160747). Western blots were performed with 20 μl input (equaling 20 μg protein), 20 μl unbound and 10–30 μl eluate.

### CRISPR/CAS9 Generation of SAF6 mutant fly lines

A SAF6 CDS deletion line was generated by targeting the 5’ and 3’ UTR by a guide RNA and recombination of a DsRed cassette with the CDS. The 5’ gRNA (5’ GATTATTAACCCAACTATCGA 3’) (primers 1 and 2, S2 Table) and 3’ guide RNA (5’ GAGTAAAGTTGTATCACCTTT 3’) (primers 3 and 4, S2 Table) were each cloned into the BbsI restriction site of pBFv-U6.2 as described before [62]. Amplicons of 500 bp spanning the guide region were generated using primers 5–8 to test if the reported Flybase genome sequence was accurate and the parent fly (BL55281) was free of SNPs.

A donor construct was created that contained a DsRed cassette flanked by 1KB homology arms that matched the upstream and downstream 1Kb DNA sequence of each guide (primers 9–12, S2 Table). The DsRed cassette and pHSG289 backbone was PCR-amplified from the pHDScarless DsRed plasmid (https://flycrispr.org/scarless-gene-editing) and the homology arms were PCR-amplified from wild-type fly genomic DNA. The PCR products were combined via HiFi DNA assembly (NEB). The plasmid was validated using Sanger sequencing.

A total of 300 embryos of parent fly (BL55821) were injected at SIMR by Paul Leal. All surviving adults were mated to w[1118]/Dp(1;Y)y[+]; noc[Sco]/SM6a. The F1 larvae were screened for DsRed signal and all adult males with DsRed positive eyes mated in single-male crosses w[1118]/Dp(1;Y)y[+]; noc[Sco]/SM6a. Male F2 progeny with DsRed balanced by Sm6A were again mated to this balancer create stable stocks of the genotype w[118];saf6CDSdelDsRed/SM6a, abbreviated as saf6[CDSdel].

### WDA allele EMS mutagenesis

The wda[EMS] stock was generated by a ethyl methanesulfonate (EMS) screen performed by Xuanying Li and Susan Abmayr. Adult males (P{neoFRT}82B ry[506]) were starved at 3–5 days post-eclosion for three hours and transferred to vials saturated with 30mM EMS, 100mM Tris-HCl and 10% sucrose. After 20 hours, the males were transferred to fresh food for three changes to clear the EMS and mated to virgin females (y,w;D[3],gl[3]/TM3,Tb[1],Ser[1]) 2–6 days post-eclosion. Progeny TM3 males were mated to WDA[4] and WDA[8] [42] and non-complementing alleles were recovered. (genotype: p{neoFRT}82B ry[5–6,wda*/ TM3,Tb, Sb]). Non-complementation crosses were performed again using deficiency stock BL25694(w[1118]; Df(3R)BSC619/TM6C, cu[1] Sb[1]. A total of 46 stocks did not complement the deficiency. The balancer was exchanged to TM3,p{w[+mC] = GAL4-tw.G}2.3,p{UAS-2EGFP}AH2.3,Sb[1],Ser[1]. Genomic DNA was obtained from homozygous embryos. Mutation in WDA was confirmed by PCR (primer 13 and 14, S2 Table) and sequencing. This allele contains a 321 bp deletion in exon1 through exon 2. Random mutations were removed by outcrossing the mutagenized chromosome for two generations to Oregon R before the phenotypic analysis.

### Germ line clones

To create germline clones, one needs a recombinant stock that contains an FRT site that is located at the same arm as where the mutated allele is positioned. (FRT82B for WDA or chromosome 3R, and FRT40A for SAF6 on chromosome 2L). The saf6 mutant allele was created in BL55821 and FRT^40A^ was recombined into stock w[118];saf6[CDSdel]/SM6a. At the same time, a heat-shock flip was introduced on the X-chromosome using y,w,HSFLP;FRT^40A^-ubiGFP/CyO since the corresponding ovo^D^ stock does not carry one (Bl2121;P{w[+mC] = ovoD1-18}2LaP{w[+mC] = ovoD1-18}2Lb {ry[+t7.2] = neoFRT}40A/ Dp(?;2) bw[D], S[1] wg[Sp-1] Ms(2)M[1] bw[D]/CyO) and balanced with y,w,HSFLP;Sco/CyO.

The actual germ line clones were created by crossing virgins from each “mutation, FRT stocks” to the corresponding ovoD males to introduce recombination. Larvae were heat-shocked for 2hr at 37°C for two days starting from the second day after hatching. Homozygous virgins were collected and used for further analyses.

### Germ-cell specific knockdown in ovaries

For each protein of interest, we tested where possible the efficacy of multiple RNAi sequences expressed from either the second or third chromosomes using pValium20 and pValium22 lines where possible. The knockdown was tested at 25 and 29°C. UAS RNAi lines to target WDA, SAF6 or TAF10b were generated by Rainbow Genetics Inc (S1 Table). To induce expression of the RNAi, UAS-RNAi flies were crossed to the maternal triple driver was chosen that expressed GAL4 maternally, with three promoters, and targets the germ line. This is stock BL31777, genotype P{w[+mC] = otu-GAL4::VP16.R}1, w[*]; P{w[+mC] = GAL4-nos.NGT}40; P{w[+mC] = GAL4::VP16-nos.UTR}CG6325[MVD1] and throughout the manuscript referred to as MTD-Gal4. Throughout the text the SAGA subunit RNAi/MTD-Gal4 genotype is abbreviated: e.g. UAS RNAi e(y)1 (BL 32345)/MTD-Gal4 is refer to as E(y)1 RNAi.

### RNA isolation

Male and female wild type flies were conditioned for three days on apple juice plates supplemented with yeast paste to fatten the ovaries. The dissections were performed in fresh and cold Shields and Sang M3 insect medium within 30 minutes. Total RNA was extracted using the QiagenEasy RNA extraction kit according to the manufacturer’s instructions, with biological triplicates for each condition. For RNA-sequencing, reads generated were 51-base-pair (bp) single-end, poly-A-selected and directional using the Illumina protocol.

### Regular ChIP in ovaries

ChIP was performed on ovaries as described before [63]. To minimize variation in the ovary ChIPs three batches of chromatin were prepared and all subunit IPs were performed on the same batch of chromatin using 20 ug chromatin to generate three biological replicates. ChIP for WDA and SAF6 was performed with 10 μg antibody, Ada2b, Spt3 and Sgf11 with the amounts described in [25]. Data was aligned to *Drosophila* genome version dm6, using bowtie with parameters -k 1 -m 3. Gene definitions utilize Ensembl 87. ChIP-seq samples were scaled to the input (sonicated whole cell extracts from ovaries) to account for differences in sequencing depth using the signal extraction scaling (SES) method and the occupancy was displayed as the log2 ratio of the IP over the input.

For each ChIP-nexus experiment, 10^7^ Kc167 cells were fixed with 1% formaldehyde in SFX culture media at room temperature for 10 minutes with rotation. Fixed cells were washed with cold PBS, incubated with Orlando and Paro’s Buffer (0.25% Triton X-100, 10 mM EDTA, 0.5 mM EGTA, 10 mM Tris-HCl pH 8.0, supplemented with 1x Protease Inhibitor) for 10 minutes at room temperature with rotation, and then centrifuged and re-suspended in ChIP Buffer (10 mM Tris-HCl, pH 8.0; 140 mM NaCl; 0.1% SDS; 0.1% sodium deoxycholate; 0.5% sarkosyl; 1% Triton X-100, supplemented with 1x Protease Inhibitor). Sonication was performed with a Bioruptor Pico for five rounds of 30 seconds on and 30 seconds off. Chromatin extracts were then centrifuged at 16000 g for 5 minutes at 4°C, and supernatants were used for ChIP.

To couple Dynabeads with antibodies, 50 μl Protein A and 50 μl Protein G Dynabeads were used for each ChIP-nexus experiment and washed twice with ChIP Buffer. After removing all the liquid, Dynabeads were resuspended in 400 μl ChIP Buffer. 10 μg antibodies were added, and tubes were incubated at 4°C for 2 hours with rotation. After the incubation, antibody-bound beads were washed twice with ChIP Buffer.

For chromatin immunoprecipitation, chromatin extracts were added to the antibody-bound beads and incubated at 4°C overnight with rotation and then washed with Nexus washing buffer A to D (wash buffer A: 10 mM Tris-EDTA, 0.1% Triton X-100, wash buffer B: 150 mM NaCl, 20 mM Tris-HCl, pH 8.0, 5 mM EDTA, 5.2% sucrose, 1.0% Triton X-100, 0.2% SDS, wash buffer C: 250 mM NaCl, 5 mM Tris-HCl, pH 8.0, 25 mM HEPES, 0.5% Triton X-100, 0.05% sodium deoxycholate, 0.5 mM EDTA, wash buffer D: 250 mM LiCl, 0.5% IGEPAL CA-630, 10 mM Tris-HCl, pH 8.0, 0.5% sodium deoxycholate, 10 mM EDTA). End repair and dA-tailing were performed using the NEBNext End Repair Module and the NEBNext dA-Tailing Module. ChIP-nexus adaptors with mixed fixed barcodes (CTGA, TGAC, GACT, ACTG) were ligated with Quick T4 DNA ligase and converted to blunt ends with Klenow fragment and T4 DNA polymerase. The samples were treated with lambda exonuclease and RecJ_f_ exonuclease for generating Pol II footprints at high resolution. After each enzymatic reaction, the chromatin was washed with the Nexus washing buffers A to D and Tris buffer (10 mM Tris, pH 7.5, 8.0, or 9.5, depending on the next enzymatic step).

After RecJ_f_ exonuclease digestion, the chromatin was eluted and subjected to reverse crosslinking and ethanol precipitation. Purified single-stranded DNA was then circularized with CircLigase, annealed with oligonucleotides complementary to the BamHI restriction site and linearized by BamHI digestion. The linearized single-stranded DNA was purified by ethanol precipitation and subjected to PCR amplification with NEBNext High-Fidelity 2X PCR Master Mix and ChIP-nexus primers. The ChIP-nexus libraries were then gel-purified before sequencing with Illumina NextSeq 500.

### TSS selection

TSSs from non-overlapping Flybase protein coding genes (fb-r56.4741) were re-annotated using CAGE-seq data from *Drosophila* melanogaster kc167 cells (modENCODE_5333) and virgin fly ovaries (modENCODE_5368). Before alignment, raw fastq files were trimmed using Cutadapt to remove the 9-nt adapter sequence (GATCAGCAG for kc167 and ACGCAGCAG for ovaries) attached to the 5’ end of reads. Adapter-removed reads were then aligned to dm6 using STAR and further processing and analysis was done with the CAGEr package from R. For TSS tag clustering (TCs) low-quality reads were removed and replicates were merged (only for Kc167 cells). Clusters with only one TSS (singletons) were discarded unless the normalized signal (TPM) was equal or above 5. TSSs within 40-bp of each other were clustered together and only clusters with at least 12 TPM from all TSS positions were considered expressed and kept for further analysis.

### Promoter type analysis

Promoter types were defined by the presence or absence of the motifs listed in S3 Table. TATA and TCT promoters were only required to contain the TATA-box and the TCT element, respectively, but were not excluded for having other elements. Conversely, DPR and housekeeping promoters were additionally filtered to not have motif elements from any other of the promoter categories. Thus, DPR promoters were allowed the presence of MTE, DPE and PB but not TATA, TCT, Ohler1/6/7 or DRE, whereas housekeeping promoters were allowed to contain Ohler1/6/7 or DRE but not TATA, TCT, MTE, DPE or PB.

DNA sequences at each promoter category (TATA, TCT, DPR and Housekeeping) were obtained from the dm6 genome and represented as heatmap. The motifs logos were generated using the R package ggseqlogo. ChIP-nexus average binding profiles (metaprofiles) were plotted in 201-bp window using the average footprints for each factor in reads per million (RPM) on the positive strand (above line) and negative strand (below line) across the different promoter groups. Signal (boxplots) were generated by calculating the total number of RPM at each promoter in a 101-bp window centered at the TSS for ChIP nexus data, whereas for ChIP-seq boxplots were generated using the maximum value in 501-bp window centered at the TSS.

### Differential contrast imaging

Most DIC images were obtained using a Nikon Ti2 motorized widefield microscope equipped with Nikon Perfect Focus technology and a sCMOS camera. However, due to COVID-19 research restrictions selected images had to be captured using an Axioplan2 wide field microscope using a 20x air objective. Multiple images were captured to cover each ovariole and merged with Photoshop using the default settings (Files➔ Automate ➔photo merge). Grey boxes have been placed behind the image to obtain equal panel sizes when required.

### Western blotting analysis

The gel filtration samples were resolved on 10% SDS-PAGE gels. The HAT assay samples were resolved on 15% SDS-PAGE gels. Gels were transferred to a PVDF membrane and blocked for one hour at 4°C in 5% milk in Tris-buffered saline (TBS) and 0.1% Tween-20. Primary antibodies were diluted in 5% milk in TBS and 0.1%Tween-20 and incubated overnight at 4°C. The following antibodies were used: Gcn5 (rabbit polyclonal, 1:1000, (GenScript antibody services, Atlanta, GA, USA anti full-length Gcn5); Ada2b (rabbit polyclonal, 1:1000; GenScript anti-amino-acid 1–330); anti-Flag- horseradish peroxidase (mouse, 1:5000; Sigma Millipore); H3 (rabbit, 1:10000; Abcam); H3K9/K14ac (rabbit, 1:1000, Sigma Millipore); Donkey anti-rabbit IgG-horseradish peroxidase (1:5000, Fisher Scientific). The rabbit TBP antibody was kindly shared by the Zeitlinger laboratory.

## Supporting information

S1 Table List of genotypes. This table includes an overview of the *Drosophila* stocks that were used in this study.
(DOCX)

S2 Table List of primers. This table includes the sequences of the PCR primers used in this study.
(DOCX)

S3 Table Motif Sequences. Promoter groups were defined based on presence or absence of the different motifs. Each motif was scanned within the start and end windows using the TSS as a reference. A positive match for either DPE_O [63] or DPE_K was considered a positive hit for a DPE element, as these are reported variants of the same motif.
(DOCX)

S1 Fig Germ line depletion of structural subunits does not affect oogenesis. (A) Spt20 RNAi ovariole. (B) Spt7 RNAi ovariole. Scale bar: 50 μM.
(TIF)

S2 Fig Genome-wide occupancy of SAGA subunits in ovary tissue intersected with RNA-seq shows that SAGA broadly binds to active promoters. The 4,000 active genes were divided into ten quantiles based on Oregon R wild type ovary RNA-seq expression levels (decreasing from left to right) and each box plot panel displays the SAGA subunit occupancy per quantile (log2 ratio over input). This shows that SAGA occupancy levels correlate with transcriptional activity.
(EPS)

S3 Fig Define ovary cell genes by promoter type. (A) The DNA sequence of each promoter type (TATA, DPE, TCT, HK) is shown as colored letters for a 101 bp window centered around the transcription start site. (B) The position weight matrix (PWM) logo for each promoter type (TATA, DPE, TCT, HK).
(EPS)

S4 Fig SAGA binding pattern at different promoter types in ovary tissue. (A) Average binding of SAGA subunits at gene promoters within each promoter group (left to right TATA, DPE, TCT and HK. (B) SAGA occupancy does not drastically change between promoter types. For each promoter group, binding occupancy at each gene is calculated as the maximum value in a 500 bp window centered at the TSS.
(EPS)

S5 Fig Define KC167 cell genes by promoter type. (A) The DNA sequence of each promoter type (TATA, DPE, TCT, HK) is shown as colored letters for a 101 bp window centered around the transcription start site. (B) The position weight matrix (PWM) logo for each promoter type (TATA, DPE, TCT, HK).
(EPS)
