# Supplementary material for: The SAGA core module is critical during Drosophila oogenesis and is broadly recruited to promoters
Source: PLoS Genet. 2021 Nov 22;17(11):e1009668. doi: 10.1371/journal.pgen.1009668 (PMC8648115; doi:10.1371/journal.pgen.1009668)
Supplement: S1 Table — This table includes an overview of the Drosophila stocks that were used in this study. (DOCX) [file pgen.1009668.s001.docx]

**S2 Table: List of primers.**

| Primer # | Name | sequence (5’ to 3’) |
| --- | --- | --- |
| Primer 1 | 5’ SAF6 guide sense | cttcgattattaacccaactatcga |
| Primer 2 | 5’ SAF6 guide anstisense | aaactcgatagttgggttaataatc |
| Primer 3 | 3’ SAF6 guide sense | cttcgagtaaagttgtatcaccttt |
| Primer 4 | 3’ SAF6 guide antisense | aaacaaaggtgatacaactttactc |
| Primer 5 | 5’ SAF6 guidesnp region sense | acagctcctcctgaatcctca |
| Primer 6 | 5’ SAF6 guidesnp region anti sense | gcgccagaggttttcatctt |
| Primer 7 | 3’ SAF6 guidesnp region sense | gcgactgttccccactcc |
| Primer 8 | 3’ SAF6 guidesnp region antisense | gtccagcgaattgagcacg |
| Primer 9 | 5’ SAF6 homology arm sense | tagaggatcctccagcaaaagacgtagc |
| Primer 10 | 5’ homology arm antisense | ctttctagggttaaaacagttggtaaataaacaac |
| Primer 11 | 3’ homology arm sense | atgtatcttattaaggtgatacaactttactcgtaataca |
| Primer 12 | 3’ homology arm antisense | cggtacccggtcgccactctgggtgcac |
| Primer 13 | WDA deletion validation sense | ttgcatcccgctgggtcgta |
| Primer 14 | WDA deletion validation antisense | gccaaatagccatcactgacg |
