# Supplementary material for: The SAGA core module is critical during Drosophila oogenesis and is broadly recruited to promoters
Source: PLoS Genet. 2021 Nov 22;17(11):e1009668. doi: 10.1371/journal.pgen.1009668 (PMC8648115; doi:10.1371/journal.pgen.1009668)
Supplement: S3 Table — Promoter groups were defined based on presence or absence of the different motifs. Each motif was scanned within the start and end windows using the TSS as a reference. A positive match for either DPE_O [63] or DPE_K was considered a positive hit for a DPE element, as these are reported variants of the same motif. (DOCX) [file pgen.1009668.s003.docx]

**S3 Table: Motif sequences.**

| **Name** | **Sequence** | **Start window** | **End window** |
| --- | --- | --- | --- |
| TATA | STATAWAWR | -40 | -20 |
| DPE_K | RGWCGTG | 20 | 50 |
| DPE_O | KCGGTTS | 20 | 50 |
| MTE | CSARCSSA | 10 | 30 |
| PB | KCGRWCG | 20 | 50 |
| DRE | ATCGAT | -70 | -1 |
| Ohler1 | YGGYCACACTR | -100 | 50 |
| Ohler6 | YRGTATWTTY | -150 | 25 |
| Ohler7 | YCAKCNCTA | -100 | 50 |
| TCT | YYCTTTYY | -10 | 10 |
