# Supplementary figures and images for: The SAGA core module is critical during Drosophila oogenesis and is broadly recruited to promoters
Source: PLoS Genet. 2021 Nov 22;17(11):e1009668. doi: 10.1371/journal.pgen.1009668 (PMC8648115; doi:10.1371/journal.pgen.1009668)

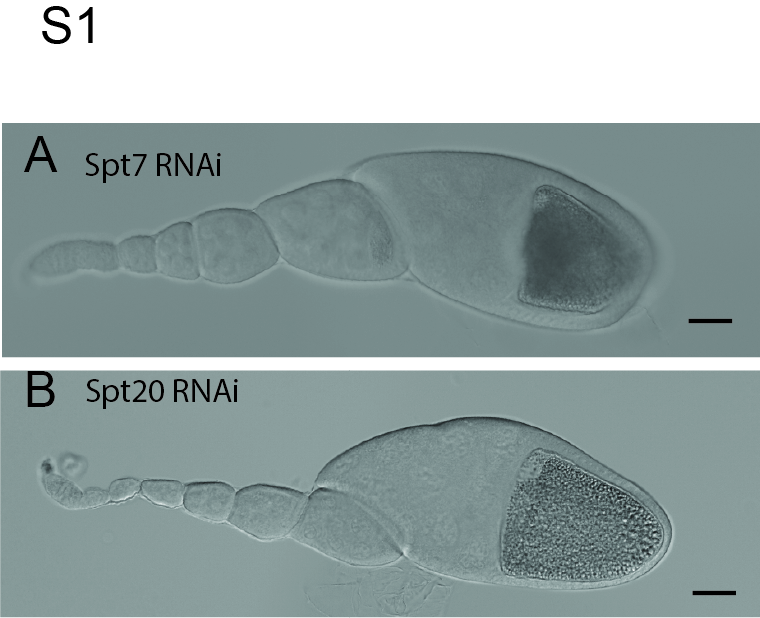

Supplement: S1 Fig — (A) Spt20 RNAi ovariole. (B) Spt7 RNAi ovariole. Scale bar: 50 μM. (TIF) [file pgen.1009668.s004.tif]
